# Glyphosate used as desiccant contaminates plant pollen and nectar of non-target plant species

**DOI:** 10.1016/j.heliyon.2022.e12179

**Published:** 2022-12-08

**Authors:** Elena Zioga, Blánaid White, Jane C. Stout

**Affiliations:** a Botany, School of Natural Sciences, Trinity College Dublin, Dublin 2, Ireland; b School of Chemical Sciences, DCU Water Institute, Dublin City University, Dublin 9, Ireland

**Keywords:** Pesticides, Herbicides, Translocation, Bees, Honey

## Abstract

Pesticide products containing glyphosate as a systemic active ingredient are some of the most extensively used herbicides worldwide. After spraying, residues have been found in nectar and pollen collected by bees foraging on treated plants. This dietary exposure to glyphosate could pose a hazard for flower-visiting animals including bees, and for the delivery of pollination services. Here, we evaluated whether glyphosate contaminates nectar and pollen of targeted crops and non-target wild plants. Oilseed rape was selected as focal crop species, and *Rubus fruticosus* growing in the hedgerows surrounding the crop was chosen as non-target plant species. Seven fields of oilseed rape, where a glyphosate-based product was applied, were chosen in east and southeast Ireland, and pollen and nectar were extracted from flowers sampled from the field at various intervals following glyphosate application. Pollen loads were taken from honeybees and bumblebees foraging on the crop at the same time. Glyphosate and aminomethylphosphonic acid (AMPA) residues were extracted using acidified methanol and their concentrations in the samples were determined by a validated liquid chromatography tandem mass spectrometry (LC-MS/MS) method. Glyphosate was detected in *R. fruticosus* nectar and pollen samples that were taken within a timeframe of two to seven days after the application on the crop as a desiccant. No glyphosate was detected when the application took place before or more than two months prior to our sampling in any of the evaluated matrices. The metabolite AMPA was not detected in any samples. To gain further insight into the potential extent of translocation within both plants and soil when a crop is desiccated using glyphosate before harvesting, and the potential impacts on bees, we recommend a longitudinal study of the presence and fate of glyphosate in non-target flowering plants growing nearby crop fields, over a period of several days after glyphosate application.

## Highlights

• Pollen and nectar were evaluated for glyphosate and AMPA residues.
• Glyphosate applied as desiccant was detected in wild plant nectar and pollen.
• No AMPA was detected in any of the samples.
• Nontarget flowering plants are a significant exposure route of bees to glyphosate.

## 1 Introduction

Glyphosate (*N*-(phosphonomethyl) glycine) is a systemic herbicide that has become the most commonly used herbicide worldwide since its commercial introduction in 1974. This is due to several properties: it is non-selective, broad spectrum, phloem-mobile, and available at low production cost (Benbrook, 2016; Duke and Powles, 2008; Ledoux et al., 2020). In Ireland, glyphosate is the most used active ingredient in arable crops (DAFM, 2016), and it is found in over 100 products approved for use (DAFM, 2020). Glyphosate is applied to agricultural fields, typically either before crop planting or after harvest, to control the growth of annual and perennial weeds (Grao et al., 2022; Silva et al., 2018). It is also used to a lesser extent as a desiccant; a pre-harvest treatment to facilitate the harvesting process by regulating plant growth and ripening (European Commission, 2022; Karise et al., 2017; Van Bruggen et al., 2018; Zhang et al., 2017). Glyphosate kills plants by inhibiting one part of the shikimate pathway, the 5-enolpyruvylshikimate-3-phosphate synthase, an essential enzyme found in plants, fungi, and some bacteria (Duke and Powles, 2008). The major metabolite of glyphosate is aminomethylphosphonic acid (AMPA) (Blot et al., 2019; Kanissery et al., 2019), and can be found both in plants and in the soil (Arregui et al., 2004; Reddy et al., 2008). Glyphosate is highly soluble and considered non-persistent, with DT_50_ values (time required for 50% dissipation of the initial concentration) of 1.1–13.7 days reported in field studies (PPDB, 2022). This is in sharp contrast to AMPA, which is classed as persistent with field study DT_50_ values of 283.6–633.1 days reported (PPDB, 2022). Considering the higher persistence of AMPA, and that it has similar toxicological significance to glyphosate (Gomes et al., 2014; Kwiatkowska et al., 2014; Woźniak et al., 2018), both compounds are considered in residue analyses and regulations (Codex Alimentarius, 2013; EPA, 2020).

In many studies, glyphosate and AMPA have been detected in all treated plant products, but also in non-target plants, the soil, animals that feed on crop products, surface and groundwaters (and the organisms that live there), the atmosphere, and humans (Benbrook, 2016; Perez et al., 2011; Van Bruggen et al., 2018). The use of glyphosate as a herbicide that is non-toxic to non-target organisms (e.g., beneficial insects, aquatic life, humans etc) and as a very efficient way for weed control is controversial, and due to its extensive use (Van Bruggen et al., 2018), there is increasing evidence for its ecotoxicological effects on non-target agroecosystem biodiversity (Cuhra et al., 2016; Kanissery et al., 2019; Yamada et al., 2009). Herbicides can impact not only their application area, but also the non-target plant species growing on the margins of that area (Russo et al., 2020). Non-target plants growing in the margins of the main crop are significant in that they provide habitat and food for pollinators in agricultural systems, including both managed honeybees (Pettis et al., 2013) and a range of wild pollinating insects (Grab et al., 2019; Park et al., 2015; Russo et al., 2016). Bees play an important role in both crop and wild plant pollination (Potts et al., 2016), but they are under threat, by the intensification of agriculture and the widespread use of various pesticide products (e.g., insecticides, fungicides, and herbicides) (McArt et al., 2017; Rundlöf et al., 2015; Woodcock et al., 2016).

Glyphosate threatens bees indirectly by reducing the availability of nutritional resources (Bohan et al., 2005; Gill et al., 2018; Sharma et al., 2018). Even though there is conflicting evidence for glyphosate's toxicity to bees, the recent meta-analytical review on papers that evaluated the effect of glyphosate on bee mortality concluded that glyphosate can be toxic to bees and highlighted the need for further evaluation of both lethal and sublethal effects in bee species other than honeybees (including stingless and solitary bees) (Battisti et al., 2021). AMPA concentrations found in beebread led to sub-lethal exposure to bees (El Agrebi et al., 2020), but the number of studies is still too low to draw any definitive conclusion on the effects of AMPA on bees (Battisti et al., 2021).

Glyphosate has been detected in various hive matrices, including pollen, nectar, honey, and honeybee larvae (Berg et al., 2018; Chamkasem and Vargo, 2017; Ferreira de Souza et al., 2020; Karise et al., 2017; Kyriakopoulou et al., 2017; Rubio et al., 2014; Thompson et al., 2014, 2019; Zhu et al., 2017; Zoller et al., 2018), and sometimes in remarkably high concentrations (Thompson et al., 2014; Kyriakopoulou et al., 2017). However, there is still a lack of information on the field realistic concentrations of glyphosate in plant derived pollen and nectar, and further data collection is required from a regulatory perspective for that specific compound (Kyriakopoulou et al., 2017). A recent review has demonstrated that systemic insecticides and fungicides may contaminate plant pollen and nectar of both target and non-target plant species, and the non-target exposure route to pesticides has been identified as more relevant for all bee species and not just honeybees (Zioga et al., 2020). Nevertheless, an accurate and complete understanding of the presence, persistence, and translocation of glyphosate residues in crop and non-crop plant pollen and nectar is still lacking (Zioga et al., 2020). Hence, in the present study, we provide more information on the concentration of glyphosate in plant derived pollen and nectar and examine whether this contamination affects crop and non-target plant species. For this, we selected two plant species (a crop and a non target wild plant) suggested by previous studies as good candidates for residue evaluation studies (Zioga et al., 2020): the oilseed rape crop species (OSR - *Brassica napus* L.) and the non-target wild plant blackberry (BAB - *Rubus fruticosus* L. agg.) that grows on the margins of the crop field and is a highly valuable food resource for flower-visiting insects (Wignall et al., 2020). Specifically, we tested whether glyphosate residues could be detected and quantified in 1. Crop nectar and pollen, 2. Wild plant nectar and pollen and 3. Bee-collected pollen.

## 2 Materials and methods

### 2.1 Field locations and sampling method

**Figure 1:**
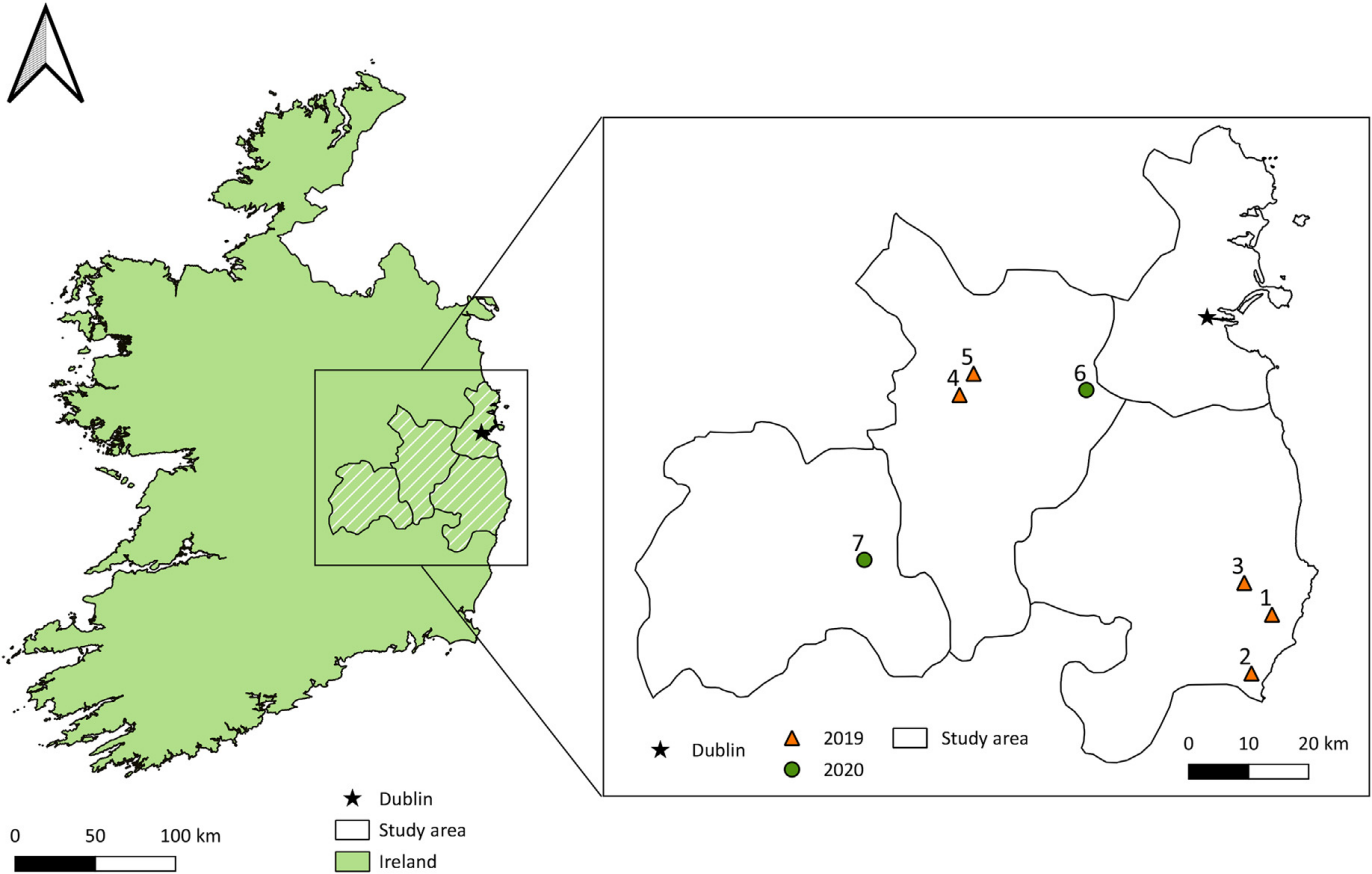
The map of the seven sampled oilseed rape fields in 2019 and 2020.

Seven fields growing winter-planted OSR (WOR, fields 1, 2, 3, and 4) and spring-planted OSR (SOR, fields 5, 6, and 7) were selected in the East and Southeast of Ireland (Figure 1) and sampled during crop flowering (March/April for WOR, June/July for SOR) in 2019 and 2020. The choice of fields was based on whether the active ingredient, glyphosate, had been applied on the crops at any stage of the cultivation period. For each field, all information related to glyphosate applications during the crop season (e.g., product applied, date and rate of application etc.) (Table S1) along with general information regarding the field (e.g., crop variety and cropping history) was recorded after interviewing the farmers (Table S2). In 2019, WOR and SOR flowers were collected for pollen and nectar extraction. That year, blackberry (BAB) flowers from plants growing on the edges of the fields where OSR crops were cultivated were also collected for pollen and nectar extraction. In 2020, SOR flowers were collected for pollen extraction. In fields 2 and 4, BAB flowers were collected twice 2–7 days after the glyphosate application on the crop. To obtain sufficient nectar and pollen for chemical analysis (100 mg and 100 μL nectar), a minimum of 1000 flowers from each plant species in each field during each sampling event were collected. Nectar was collected into individual eppendorf tubes for each sample with a microcapillary tube and pollen was retrieved from the anthers, after incubation in 37 °C for 24 h (e.g., see Botías et al., 2015). In 2020, honeybee and bumblebee workers foraging for pollen on the crop were also collected (Table S2). To acquire sufficient pollen for chemical analysis, a minimum of 20 honeybees and 15 bumblebees observed actively foraging on the crops, and carrying relatively large amounts of corbicular pollen, were randomly collected from each field. The bees were caught by net and placed in plastic vials in coolers before being transferred to the lab. All samples were weighed and stored at -25 °*C prior* to analysis. Pollen pellets collected from each individual bee species collected in every site were combined and palynological analysis was performed. 500 grains of pollen were counted for each slide and identified based on their morphology, using a standard key (Crompton and Wojtas, 1993), pollen reference samples collected on the sampling fields, and online pollen reference collections (e.g., Chadwick et al., 2019).

### 2.2 Pesticide residue analysis

#### 2.2.1 Chemicals and reagents

Certified analyte standards of glyphosate and AMPA, formic acid, ammonium bicarbonate (HNH_4_CO_3_), Millipore Millex syringe filters with hydrophobic PTFE membrane (pore size 0.22 μm and 20 mm diameter) and low-adsorption LC-MS certified vials were obtained from Sigma Aldrich, Ireland. The internal standards (IS) Glyphosate-2-^13^C,^15^N and AMPA-^13^C,^15^N were purchased from LGC, Ireland. The HILICpak VT-50 2D column 2.1 × 100 mm and 5 um particle size using HILICpak VT-50G 2A 2 × 10 mm were all purchased from Shodex, United States. All pesticide standards were > 98% compound purity and deuterated standards > 99% isotopic purity. Ultrapure water was generated using ELGA Purelab Ultra SC MK2 (ELGA, UK), and LC-MS grade acetonitrile (ACN) and methanol (MeOH) were obtained from Sigma-Aldrich, Ireland. Individual standard pesticide (native and deuterated) stock solutions (1 mg/mL) were prepared in ultrapure water. An additional IS mixture of the deuterated pesticides at 1000 ng/mL was also prepared. Calibration solutions were freshly prepared from the stock solutions. All stock solutions were preserved at -25 °C in dark conditions.

#### 2.2.2 Nectar extraction

100 μL of nectar was placed into a 15 mL PTFE centrifuge tube. A solution of H_2_O/ACN (70:30) was added so that the volume of the final extract was 1 mL. The nectar sample was then homogenized using a vortex and centrifuged at 2500 rpm for 5 min. The solution was filtered through 20 mm PTFE hydrophilic syringe filters (Sigma-Aldrich) into an LC-MS vial.

#### 2.2.3 Pollen extraction

A modified protocol of the Quick Method for the Analysis of numerous Highly Polar Pesticides in Foods of Plant Origin via LC-MS/MS (QuPPe-Method) was used for pollen, as suggested by the (European commission, 2021). Briefly, 100 mg of pollen was weighed in a 50 mL PTFE centrifuge tube to which 5 mL of acidified MeOH (1% v/v formic acid) was added. The tube was vortexed for 1 min. The tube was centrifuged at 4500 rpm for 10 min. The supernatant was transferred into a 15 mL PTFE centrifuge tube and was placed in -25 °C for 1 h. An aliquot of the supernatant was mixed with H_2_O/ACN (70:30). The solution was filtered through 20 mm PTFE hydrophilic syringe filters (Sigma-Aldrich) into an LC-MS vial.

#### 2.2.4 LC-MS/MS analysis

The analysis of the two compounds in both nectar and pollen was performed on an Agilent 1290 Infinity II Multisampler LC System coupled to an Agilent 6470 Triple Quadrupole mass spectrometer (LC-MS/MS). Compound separation was achieved on a HILICpak VT-50 2D column (2.1 × 100 mm and 5 um particle size, Shodex) using a column oven at 30 °C and a flow rate of 0.1 mL/min. Mobile phase A consisted of 50 mM ammonium bicarbonate in H_2_O and mobile phase B comprised of pure ACN. A 70% mobile phase A elution programme was used, and the total time of the LC-MS/MS analysis was 25 min. The injection volume was 20 μL. The ion spray voltage was set at -3000 V for negative ionization. Source temperature was set at 300 °C. Nitrogen was used as a curtain gas (8 L/min), nebulizer gas (30 psi), sheath gas flow (12 L/min), and sheath gas temperature was 350 °C. Agilent Mass Hunter Workstation Data Acquisition Version 10.0 software was used to control LC-MS/MS system and for data acquisition. Quantitative and qualitative analysis was carried out with Agilent Mass Hunter Workstation Qualitative Analysis software version 10.0, based on two most abundant precursor ion and product ion MRM transitions, and their characteristic retention time. The compound-specific LC-MS/MS retention times (Rt), quantifying transition ions (Q) and qualifier transition ions (q) for glyphosate, AMPA and their ISs are shown in Table S3.

#### 2.2.5 Method validation

Validation experiment was carried out according to requirements of Document No. SANTE/12682/2019 guidance (Table S4) (European Commission, 2020a, European Commission, 2020b). To prepare the pollen matrix-matched calibration, pollen free from pesticide residues was used as blank. For the nectar matrix-matched calibration, a nectar surrogate was created by mixing 6 g of glucose and 3 g of fructose in 25 mL of ultrapure water (Martel et al., 2013).

#### 2.2.6 Risk evaluation

For evaluating the potential risk that pesticide residues detected in pollen and nectar pose specifically to honeybees, the BeeREX model was used (Thompson 2021; US EPA, 2014), as previously described in (Rondeau and Raine, 2022).

## 3 Results and discussion

**Figure 2:**
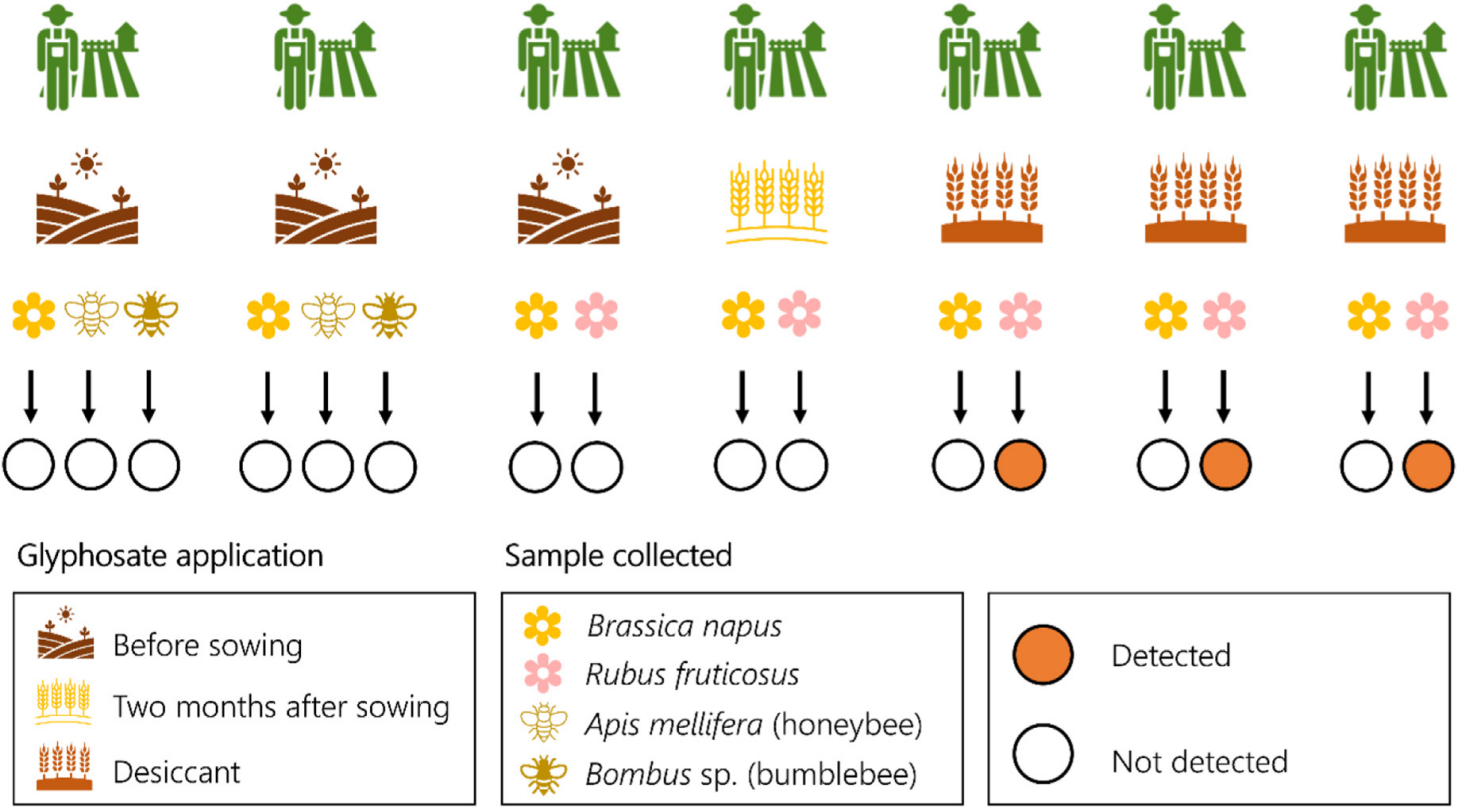
Application timing of the glyphosate product in the seven fields and the respective detections in the collected samples.

**Figure 3:**
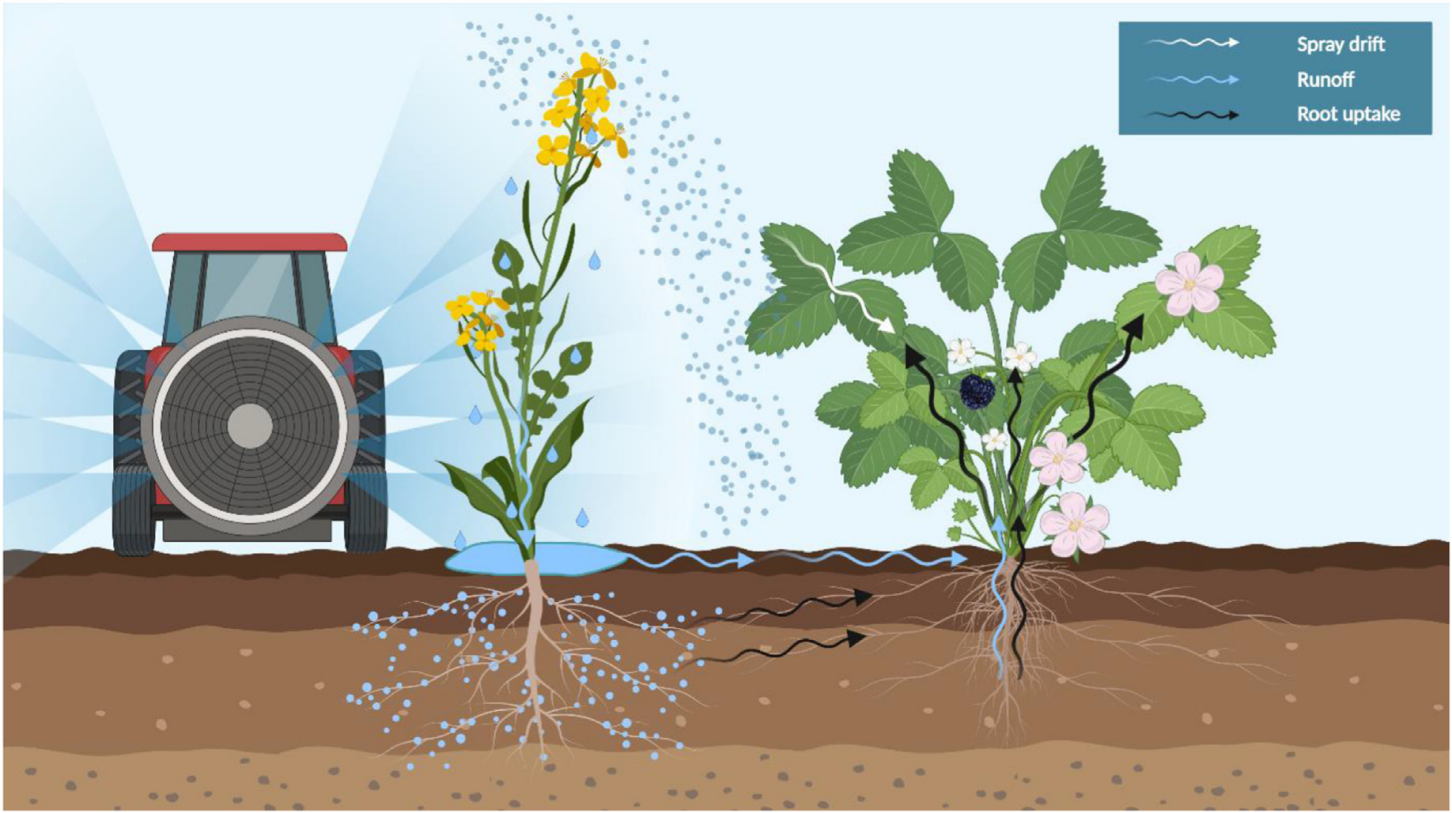
Potential glyphosate translocation pathways in soil and target and non-target plant species. Created with BioRender.com.

Residues of the active ingredient were detected in BAB pollen and nectar from three WOR fields sampled in 2019 (field 1, 2 and 4) (Table 1, Figure 2). The detections in BAB pollen and nectar originate from fields where the glyphosate product was applied as a desiccant within one week of sampling. On the other hand, when used as a pre- or post-emergence spray, no residues were detected in nectar and pollen of the crop, or the wild plant (Figure 2) sampled more than 2 months after spraying. In crop related matrices, residues increase in grain, fodder, and oil when glyphosate is used as a desiccant before harvest (Cessna et al., 1994, 2000; FAO, 2006; McNaughton et al., 2015; Zhang et al., 2017). Residues of compounds used as desiccants have been previously reported in wild plants (Vahl et al., 1998), in the environment (e.g., water) (Zhao et al., 2013), and even honeybee matrices like honey and comb pollen (Erban et al., 2017), suggesting potential exposure on non-target organisms. Here, we report for the first-time glyphosate residues in nectar and pollen of a non-target wild plant species growing in the margins of the main crop area (0–1 m distance), when the herbicide product is applied as desiccant to facilitate the harvesting process. It is suggested that off-target herbicide drift can adversely affect wild plant species on the margins of the crop fields (Gove et al., 2007; Marrs et al., 1993), changing the interactions between non-target plants and flower-visiting insects (Russo et al., 2020). Research has demonstrated that non-target movement of glyphosate during application can be up to 10% of the applied rate (Al-Khatib and Peterson, 1999; Ellis, J.M.; Griffin, 2002), and as little as 1–5% of the recommended application rate can negatively impact the mutualistic interactions with pollinators (Dupont et al., 2018). Although through our study it is difficult to determine how glyphosate contamination occurred in pollen and nectar of a non-target wild plant species, it is very important to identify the potential contamination pathways that may have caused this off-target plant contamination. Generally, glyphosate applied as foliar spray for weed control can potentially reach unintended areas and non-target plant tissues through three main pathways: 1) spray drift, 2) runoff, and 3) root uptake (Kanissery et al., 2019; Laitinen et al., 2007; Marrs et al., 1989; Russo et al., 2020) (Figure 3).

**Table 1:** Glyphosate residues detected in oilseed rape crop and wild *Rubus fruticosus* pollen and nectar, and honeybee and bumblebee collected pollen.

Field	Glyphosate residues (μg/kg)				
	Oilseed rape			Blackberry	
	Flower Pollen	Flower Nectar	Bee pollen	Flower Pollen	Flower Nectar
1^a^	-	< LOD∗	-	-	96.8 ± 4.4
2^b^	-	< LOD∗	-	< LOD	135.4 ± 0.4^1^
			-	-	205.7 ± 6.8^2^
3	< LOD	< LOD	-	< LOD	< LOD
4^c^	-	< LOD^1^∗	-	< LOQ	< LOQ^1^
	-	< LOD^2^∗	-	-	187.3 ± 9.2^2^
5	< LOD	< LOD	-	< LOD	< LOD
6	< LOD	-	< LOD	-	-
7	< LOD	-	< LOD	-	-

∗ This symbol refers to samples that were collected before the pesticide application on the crop. The dash (-) indicates that the respective samples were not evaluated for glyphosate residues. ^**1,2**^ The numbers refer to the first and second sampling performed on fields 2 and 4 for the respective samples. ^a,b,c^ The *R. fruticosus* flower sampling of these fields was performed after two (field 1), after three and five (field 2), and after two and seven days (field 4) from the application of glyphosate on the crop.

Spray drift is the movement of droplets of pesticide spray away from the target application area, caused by wind (Ochoa and Maestroni, 2018). This contamination pathway is dependent on several factors, some of which include wind intensity and direction, droplet size, temperature, and height of spraying nozzle (Ochoa and Maestroni, 2018). Foliar application of glyphosate contaminated non-target plants with the active ingredient for at least four weeks after the treatment (Neuman et al., 2006). Glyphosate spray drift may contaminate plant pollen and nectar directly, or by reaching the foliage of non-target plants, where it can be absorbed by the plant leaves other green tissues via diffusion (Duke and Powles, 2008), and be translocated to the entire plant, accumulating preferentially in young growing tissues (Franz et al., 1997). In our study, when glyphosate was applied as desiccant, the farmers advised that product application directions provided by the manufacturer were used, and the suggested measures to avoid spray drift were followed (e.g., zero to mild wind, spray boom as low as possible, no spraying close to the field boundaries, spraying characteristics according to the label recommendations etc.) (HSE, 2022). The absorption and translocation of glyphosate inside the plant may be influenced by the formulation, as those containing surfactants (and adjuvants) have a higher rate of absorption compared to glyphosate water solutions (Doublet et al., 2009), increasing the uptake and translocation of glyphosate in plants. This should be considered in higher tier risk assessment studies, when decisions should be made about whether the active ingredient or the commercial formulation should be sprayed on the evaluated plants. In our study, field 4 only, an adjuvant was applied as pod sticker (i.e., a product designed to prevent pod shatter by coating the crop with a thin film of polymer) at the same time as the glyphosate product application.

Although glyphosate is typically sprayed onto plant foliage, some chemical can reach the soil through spray-drift, by being washed off plant surfaces during precipitation (Al-Kathib and Peterson, 1999; Ellis and Griffin, 2002; Kremer and Means, 2009; Saunders and Pezeshki, 2015), by exudation from roots or death and decomposition of treated plant residues (Kanissery et al., 2019; Laitinen et al., 2007; Mamy et al., 2016; Neumann et al., 2006; Tesfamariam et al., 2009; Von Wiren-Lehr et al., 1997). Glyphosate and AMPA display similar behavior in terms of sorption and degradation (Feng and Thompson, 1990; Simonsen et al., 2008; Bento et al., 2016), as they strongly sorb to soil minerals (Sprankle et al., 1975; Hance, 1976; Roy et al., 1989; Piccolo and Celano, 1994; Borggaard and Gimsing, 2008; Sheals et al., 2002; Gimsing and Borggaard, 2002; Gimsing et al., 2004, 2007; Veiga et al., 2001; Vereecken, 2005), accumulating in the top layer of soils (depth of 0–15 cm) (Bento et al., 2019; Kanissery et al., 2019; Silva et al., 2018). In agricultural settings, repeated glyphosate applications increase the risk of glyphosate and AMPA accumulation in topsoil (Benbrook, 2016), considering that glyphosate and AMPA may persist in soil for long periods, under certain conditions (e.g., dry soil, low temperatures, soils with strong adsorption capacity etc.) (Bento et al., 2019; Kanissery et al., 2019). However, absorbed glyphosate and AMPA can be desorbed under certain conditions such as the addition of fertilizers (Borggaard and Gimsing, 2008; Gimsing et al., 2004; Simonsen et al., 2008; Zhou et al., 2010), or heavy rain events shortly after glyphosate application, increasing the risk of glyphosate being transported, deposited, and probably accumulated in nearby areas (Bento et al., 2019; Botta et al., 2009; Candela et al., 2010; Degenhardt et al., 2012; Gjettermann et al., 2009; Hanke et al., 2010; Luijendijk et al., 2003, Peruzzo et al., 2008; Shipitalo et al., 2008; Stone and Wilson, 2006; Styczen et al., 2011; Todorovic et al., 2014; Ulén et al., 2014; Vereecken, 2005; Yang et al., 2015a, b). Glyphosate and AMPA adsorbed to solid particles suspended in water (Rügner et al., 2014; VandeVoort et al., 2013) is the main mode of offsite contamination via runoff (Yang et al., 2015b), causing a high increase to the off-site contamination levels immediately after glyphosate application (Edwards et al., 1980; Saunders and Pezeshki, 2015). In this study, during the *R*. *fruticosus* flower collection sporadic heavy rainfalls were observed throughout the sampling period, but no fertilizer was applied between the timing of glyphosate application and the flower sampling.

Glyphosate translocates within plants, accumulates in roots, and is eventually released into the rhizosphere by the root exudates (Coupland and Casely, 1979; Kanissery et al., 2019; Laitinen et al., 2007; Neumann et al., 2006). From there, it may be reabsorbed by the treated crop or nontreated nearby plants through their root system (Kanissery et al., 2019). Once inside the plant, glyphosate may be transported within the plant xylem in the apoplastic pathway or enter the phloem and get transported to metabolic sinks via the symplastic pathway (Franz et al., 1997). For both foliar and root uptake, glyphosate translocation may be basipetal or acropetal (upwards and downwards), moving toward various tissues, such as meristems, leaves flowers, and fruits (Clua et al., 2012; Dewey, 1981; Duke, 1988; Franz et al., 1997; Giesy, 2000; Shaner, 2009; Tong et al., 2017; Wagner et al., 2003; Walker and Oliver, 2008). There is previously reported evidence of non-target plant contamination through the root system within a two-day period and with an increasing rate for a six-day period (Neuman et al., 2006). BAB plants have a perennial rooting system that comprises of a main vertical root which may grow until 4 m (depending on the soil type) and many secondary roots that grow horizontally to the main root for 30–60 cm. There are also several thin roots on the secondary roots in all directions (NSW Department of Primary Industries Weed Management Unit, 2009). Given the growing distance of the BAB plants in the present study (0–1 m), it is possible that BAB plants started absorbing glyphosate through their rooting system right after the crop spraying. Glyphosate can enter in plants through the roots, or shoots emerging from the root or the trunk (Sharma and Singh, 2001). As glyphosate is stable and not immediately metabolized in many plant species, substantial amounts can be extensively translocated to regions of active growth and accumulate, particularly in young tissues (Reddy et al., 2004). Glyphosate reaches any actively growing tissue or organ (Duke and Powles, 2008), so it may reach pollen. However, it has been suggested that lipophilic pesticides tend to accumulate in pollen, as opposed to hydrophilic compounds (Raimets et al., 2020). Glyphosate is a hydrophilic compound, and it is beyond the scope of this work to investigate whether its presence in the pollen samples in this study was as a result of translocation through the plant from the soil or instead arose through a contamination event, e.g. drift. The physicochemical properties and high solubility of glyphosate in water (PPDB, 2022) enable it to be translocated via the phloem to the same tissues that are metabolic sinks for sucrose (Siehl, 1997). Even though a detailed pathway has not yet been elucidated, nectar, represents secretions of phloem sap for most species (Heil, 2011). Hence, this is a potential pathway of translocated glyphosate to nectar and the increased concentration of glyphosate residues in BAB nectar could be a result of the compound's high solubility in water in combination with the rainfalls that occurred after the pesticide application and between the two different sampling dates at those fields. The difference in pollen and nectar concentrations or presence/absence could be linked to the different translocation pathways of glyphosate in pollen and nectar. Whether nectaries and anthers are supplied by phloem or xylem seems to play an important role in the final concentrations of residues in those matrices (Cowles and Eitzer, 2017). However, more research is required to elucidate the factors that affect the translocation of a compound inside the plant until it reaches pollen and/or nectar, with emphasis on the compound physicochemical properties (Gierer et al., 2019) and the plant species evaluated, as translocation patterns are plant species dependent (ATSDR, 2020).

No AMPA residues were detected in any of the 25 samples evaluated (< LOD). Also, no residues of glyphosate were detected in any of the OSR pollen and nectar samples of all seven fields, nor in any of the honeybee and bumblebee collected pollen from the two fields sampled in 2020 (field 6 and 7). Most of these samples were collected either prior to glyphosate application (e.g., OSR flowers of fields 1, 2 and 4) or more than two months (> 75 days) after the glyphosate application on the field (e.g., OSR and BAB flowers of fields 3, 5, 6 and 7), and the only exception was the BAB pollen of field 2 where sampling was performed three days after the application, but no residues were detected. Glyphosate and AMPA are both adsorbed to clay and organic matter particles (Banks et al., 2014; Okada et al., 2016; Tang et al., 2019). Once adsorbed their degradation is very slow and both compounds are characterized as persistent in soils (EFSA, 2015). The rates of glyphosate and AMPA degradation are highly variable, ranging from a few days up to one or two years (Saunders and Pezeshki, 2015; Silva et al., 2018; Yang et al., 2015a), with AMPA being more persistent than glyphosate (Bento et al., 2016). Degradation seems to increase in soils with increasing pH, high percentage in organic matter, higher temperatures, well-drained soil systems (Saunders and Pezeshki, 2015), increasing concentrations of inorganic soil phosphate, and decreasing mineral concentrations (Glass, 1987; Gerritse et al., 1996; Piccolo et al., 1994; Plimmer et al., 2004; Smith and Oehme 1992; Sprankle et al., 1975). Hence, a combination of various field characteristics (e.g., soil properties, plant species etc.) and the impact of external factors (e.g., climate, fertilization management, chemical interventions etc.) may be the reason why no AMPA was detected in OSR pollen and nectar in the present study. Glyphosate dissipation rate in plant matrices is estimated to be within the range of 2.2–17.0 days (PPDB, 2022) and metabolism of glyphosate within the plant occurs slowly (Doublet et al., 2009; Reddy et al., 2004; Smith and Oehme, 1992). Hence, the absence of AMPA in the BAB pollen and nectar may be attributed to the fact that glyphosate was not yet breaking down to an extent that could be detected with our method.

The fact that glyphosate and AMPA cannot be analyzed in the same multiresidue analysis along with other pesticides due to their chemical characteristics (e.g., high polarity) (Pareja et al., 2019; Vicini et al., 2021), adds an extra cost and complexity to analysis and may account for the absence of this compound in the list of the screened pesticides in most studies evaluating pesticide residues in honeybee related matrices (Toselli and Sgolastra, 2020). However, when it is evaluated, glyphosate is among the pesticides that are frequently detected in beehive matrices (Berg et al., 2018). Residues have been detected in larval honeybees (up to 19.5 mg/kg) (Thompson et al., 2014), in beebread (47.2–58.4 μg/kg) (Bergero et al., 2021; El Agrebi et al., 2020), brood (34 μg/kg), and nurse bees (289 μg/kg) (Raimets et al., 2020). It was found that honeybees and bumblebees can be exposed to high concentrations of glyphosate when foraging (up to 629.0 mg/kg) (Thompson et al., 2014, 2022), while residues of both glyphosate and AMPA were detected in stingless bees (< 50 μg/kg) (Guimarães-Cestaro et al., 2020). Regarding AMPA, no residues were found in honey and beeswax, but in beebread, the maximum AMPA concentration reached 250 μg/kg (El Agrebi et al., 2020). In our study, we did not detect any AMPA, while the glyphosate concentrations in BAB nectar ranged from < 25 μg/kg (< LOQ) to 205.7 μg/kg with an average value of 156.3 μg/kg and in BAB pollen < 35 μg/kg (< LOQ). The glyphosate values we detected in BAB nectar are much lower than the values detected in bee collected pollen and nectar from recently treated plants, but they are higher than those detected in beebread.

In honeybee collected pollen and nectar, and bumblebee collected pollen from plants that were directly sprayed with glyphosate, concentrations declined over time after the initial spray application (Thompson et al., 2014, 2022). On the other hand, the glyphosate residues detected in BAB nectar of fields 2 and 4 where the herbicide was applied as a desiccant to the crop, seem to increase over time. Even though the number of data points is too low to draw definite conclusions, the number of flowers collected per sample in each field (> 1000 flowers) represents a big sample area in each field. Yet, it remains uncertain whether contamination of non-target plant pollen and nectar may lead to higher concentrations of glyphosate after several days of the pesticide application on the crop and this is something that should be further evaluated. To elucidate the contamination pathway, we recommend an experiment specifically designed for this purpose, where the physiology of the non-target plants is also considered (e.g., the duration of the open flower present on the plants while spraying etc.).

The Risk Quotient values did not exceed the thresholds set by both US EPA and EFSA for the foragers and in-hive honeybees risk assessment (Table S5). However, our knowledge of the effects of glyphosate on honeybees is scarce and there is significant lack of information on how glyphosate affects the roughly 20,000 species (Orr et al., 2020) of wild bees (Belsky and Joshi, 2020; Franklin and Raine, 2019; Seide et al., 2018). Most of the toxicity studies have focused on honeybees (*Apis mellifera*) with only a few testing the impacts on other bee species (Abraham et al., 2018; Seide et al., 2018). Yet, when other bee species are tested, the herbicide glyphosate is found to be highly toxic (Gomes, 2017; Seide et al., 2018) or has negative effects on their physiological behavior (e.g., wild bee flight ability) (Gomes, 2017).

In recent years, the worldwide intensive use of glyphosate and its accumulation in the environment and edible products has raised major concerns about noxious side effects of glyphosate and AMPA on plant, animal, and human health (Van Bruggen et al., 2018, 2019, 2021). Based on several publications on potential chronic side effects of glyphosate-based products on human health the World Health Organization reclassified the herbicide glyphosate as probably carcinogenic to humans in 2015 (International Agency, 2015). This implies that honey contaminated with glyphosate residues above the established MRLs may pose a hazard for human health (Ledoux et al., 2020). At the same time, the sub-lethal effects caused by glyphosate on honeybees could result in the reduction of pollination services, which would impact the worldwide food production (Ledoux et al., 2020). The concentrations of glyphosate and AMPA in plants vary widely, but the maximum residue limits (MRLs) for glyphosate are set to 0.1–40 mg/kg for most plant products aimed for human consumption (Codex Alimentarius, 2013; Cuhra, 2015; EPA, 2020; European Commission, 2020a, European Commission, 2020b; FAO, 2006), and at 50 μg/kg for honey (European Commission, 2013). Glyphosate has been detected in honey worldwide with concentrations reaching up to 300 μg/kg (Berg et al., 2018; Bergero et al., 2021; Chamkasem and Vargo, 2017; Ferreira de Souza et al., 2020; Pareja et al., 2019; Raimets et al., 2020; Rubio et al., 2014; Thompson et al., 2019; Zoller et al., 2018). A European analysis of several honey samples and other apicultural products in 2018 identified glyphosate among the 30 most quantified compounds with residues often exceeding the MRLs for honey (EFSA, 2020). In a recent study, Pareja et al. (2019) found that glyphosate was detected in more than 81% of tested honey samples and 41% were above the European MRL for honey. In our study we found an average glyphosate concentration of 156.3 μg/kg in BAB nectar, a value that also surpasses the European MRL for honey. This value is relevant to honey MRL because inside *Apis* colonies, after honeybees collect pollen and/or nectar from contaminated sources, glyphosate could further concentrate because nectar is evaporated to make honey and incorporated in bee bread (Seeley, 1995). Values of glyphosate in honey samples may vary depending on plant species and environmental conditions (Farina et al., 2019). In honey collected close to OSR crops glyphosate residues were prevailing upon the rest pesticide compound residues (Karise et al., 2017). In our study we detected glyphosate residues only in wild plant pollen and nectar and not in honeybee and bumblebee collected pollen, which based on our palynological analysis it was mainly collected from the crop, but after several months of the pesticide application. Considering that in Europe the application of glyphosate does not take place in flowering OSR crops (since genetically modified crops are not allowed; Van Bruggen et al., 2018), in combination with the results of the present study, it would be worth evaluating whether glyphosate residues found in honey come from the actual crops, or if their presence in honey is a result of non-target contamination of wild plants.

## 4 Conclusion

Our findings suggest that offsite transport of glyphosate used as desiccant, contaminates pollen and nectar of non-target wild plant species. Based on the present knowledge of glyphosate's behavior in environmental matrices (e.g., plants and soil), this transport could be attributed to spray-drift, runoff, and/or root uptake, and is depended on several environmental factors, field related characteristics and agricultural practices. Bee species can be exposed to high glyphosate residues in the environment and honeybees can transfer these residues into their hives, contaminating honey at concentrations exceeding the established European MRLs for honey and posing a hazard to human health. Knowledge of glyphosate toxicity should be expanded to more wild bee species and respective LD_50_ and LDD_50_ values should be established. With our results we identified pollen and nectar of non-target wild plant species as an exposure route of bees to glyphosate, and we raise concerns about whether the high levels of contamination of honeybee products identified in the literature originate from the crops or are a result of non-target wild plant contamination. In advance of, and to inform the upcoming renewal of market authorization for glyphosate in the European Union, we recommend the immediate investigation of glyphosate as desiccant before harvesting crops, to elucidate the behavior of glyphosate residues in soil and non-target flowering plants growing near crop fields, over a period of several days after the desiccant spraying.

## Declarations

### Author contribution statement

Elena Zioga: Conceived and designed the experiments; Performed the experiments; Analyzed and interpreted the data; Wrote the paper.

Blánaid White; Jane C. Stout: Conceived and designed the experiments; Contributed reagents, materials, analysis tools or data; Wrote the paper.

### Funding statement

This work was supported by Irish Department of Agriculture Food and the Marine [17/S/232].

### Data availability statement

Data included in article/supp. material/referenced in article.

### Declaration of interests statement

The authors declare no conflict of interest.

### Additional information

No additional information is available for this paper.
