# Supplementary material for: Glyphosate used as desiccant contaminates plant pollen and nectar of non-target plant species
Source: Heliyon. 2022 Dec 8;8(12):e12179. doi: 10.1016/j.heliyon.2022.e12179 (PMC9755368; doi:10.1016/j.heliyon.2022.e12179)
Supplement: Supplementary material [file mmc1.docx]

**Supplementary material**

**Table S1.** Information on glyphosate-based product applications on the seven sampled oilseed rape fields.

| **Field** | **Date** | **Method** | **Product** | **Concentration** | **Application rate** | **Formulation type** |
| --- | --- | --- | --- | --- | --- | --- |
| 1 | 11-Jul-19 | Spray | Roundup Gold 450® | 450 g/L | 1.5 L/ha | Concentrate |
| 2 | 10-Jul-19 | Spray | Roundup PowerMax® | 720 g/kg | 2 kg/ha | Granule |
| 3 | 01-Oct-18 | Spray | Roundup Gold 450® | 450 g/L | 1.5 L/ha | Concentrate |
| 4 | 16-Jul-19 | Spray | RoundUp PowerMax® | 720 g/kg | 1.7 kg/ha | Granule |
| 5 | 09-Mar-19 | Spray | Mizr XL® | 360 g/L | 2.5 L/ha | Concentrate |
| 6 | 10-Mar-20 | Spray | Mizr XL® | 360 g/L | 2.7 L/ha | Concentrate |
| 7 | 18-Mar-20 | Spray | Mizr XL® | 360 g/L | 2.7 L/ha | Concentrate |

**Table S2.** Information on the characteristics of the seven fields, the temperature, the sampling date, and the plant species sampled.

| **Field** | **County** | **Area (ha)** | **Crop variety** | **Crop Type** | **Sowing date** | **Adjacent fields** | **Temp (˚C)** | **Sampling date** | **Matrix evaluated** |
| --- | --- | --- | --- | --- | --- | --- | --- | --- | --- |
| 1 | Wicklow | 8.6 | DK exception | Winter | 27-Aug-18 | Grass | 4 | 31-Mar-19 | OSR nectar |
|  |  |  |  |  |  |  | 17 | 13-Jul-19 | BAB nectar |
| 2 | Wicklow | 7.5 | Phoenix CL | Winter | 24-Aug-18 | Crop | 5 | 10-Apr-19 | OSR nectar |
|  |  |  |  |  |  |  | 17 | 13-Jul-19 | BAB pollen & nectar |
|  |  |  |  |  |  |  | 19 | 15-Jul-19 | BAB nectar |
| 3 | Wicklow | 14.6 | DK exception | Winter | 21-Aug-18 | Crop | 8 | 06-Apr-19 | OSR pollen & nectar |
|  |  |  |  |  |  |  | 15 | 06-Jul-19 | BAB pollen & nectar |
| 4 | Kildare | 13.1 | DK exception | Winter | 30-Aug-18 | Grass | 7 | 11-Apr-19 | OSR nectar |
|  |  |  |  |  |  |  | 12 | 11-May-19 | OSR nectar |
|  |  |  |  |  |  |  | 18 | 18-Jul-19 | BAB pollen & nectar |
|  |  |  |  |  |  |  | 20 | 23-Jul-19 | BAB nectar |
| 5 | Kildare | 10.6 | Hybrid | Spring | 06-Apr-19 | Crop | 20 | 04-Jul-19 | OSR pollen & nectar |
|  |  |  |  |  |  |  | 20 | 09-Jul-19 | BAB pollen & nectar |
| 6 | Kildare | 8.4 | Hybrid | Spring | 09-Apr-19 | Crop | 17 | 15-Jun-20 | OSR pollen |
|  |  |  |  |  |  |  |  |  | Honey bee pollen |
|  |  |  |  |  |  |  |  |  | Bumblebee pollen |
| 7 | Laois | 2.1 | Hybrid | Spring | 09-Apr-19 | Crop | 24 | 01-Jun-20 | OSR pollen |
|  |  |  |  |  |  |  |  |  | Honey bee pollen |
|  |  |  |  |  |  |  |  |  | Bumblebee pollen |

**Table S3.** The compound-specific LC-MS/MS retention times (Rt), quantifying transition ions (Q) and qualifier transition ions (q) for glyphosate, AMPA and their internal standards.

| **Compound** | **ESI mode** | **Q (m/z)** | **CE** | **q (m/z)** | **CE** | **Rt (min)** |
| --- | --- | --- | --- | --- | --- | --- |
| Glyphosate | (-) | 168>150 | 8 | 168>63 | 32 | 20.086 |
| AMPA | (-) | 110>79 | 36 | 110>63 | 20 | 6.492 |
| **Internal standards** |  |  |  |  |  |  |
| Glyphosate ^15^N | (-) | 171>63 | 24 |  |  | 20.018 |
| AMPA ^15^N | (-) | 112>63 | 20 |  |  | 6.490 |

**Table S4.** The parameters evaluated for the nectar and pollen method validation.

|  | **Nectar** |  |  |  |  | **Pollen** |  |  |  |  |
| --- | --- | --- | --- | --- | --- | --- | --- | --- | --- | --- |
|  | **LOD (ppb)** | **LOQ (ppb)** | **Linearity (r^2^)** | **Mean recovery (%)** | **RSD (%)** | **LOD (ppb)** | **LOQ (ppb)** | **Linearity (r^2^)** | **Mean recovery (%)** | **RSD (%)** |
| Glyphosate | 8.0 | 25.0 | 0.999 | 101.9 | 5.7 | 12.0 | 35.0 | 0.997 | 107.0 | 6.1 |
| AMPA | 8.0 | 25.0 | 0.999 | 98.1 | 6.6 | 12.0 | 35.0 | 0.999 | 99.4 | 4.7 |

**Table S5.** The maximum residues detected in plant pollen and nectar, the honey bee oral acute (LD_50_s) and chronic (10-day LDD_50_) toxicity data for glyphosate, and the estimated acute and chronic Risk Quotients for foragers and in-hive honey bees.

|  |  |  |  |  | **Risk Quotient** | | | |
| --- | --- | --- | --- | --- | --- | --- | --- | --- |
|  | **Maximum residues (μg/kg)** | |  |  | **Acute** | | **Chronic** | |
|  | **Pollen** | **Nectar** | **Oral honey bee LD_50_ (μg/bee)** | **Chronic 10 LDD_50_ (μg/bee/day)** | **Foragers** | **In-hive** | **Foragers** | **In-hive** |
| **Glyphosate** | 17.5 | 205.7 | 104.0 | 179.0 | 0.0006 | 0.0003 | 0.0003 | 0.0002 |
